# Replication Restriction of Influenza A(H5N1) Clade 2.3.4.4b Viruses by Human Immune Factor, 2023–2024

**DOI:** 10.3201/eid3101.241236

**Published:** 2025-01

**Authors:** Jakob Ankerhold, Susanne Kessler, Martin Beer, Martin Schwemmle, Kevin Ciminski

**Affiliations:** University of Freiburg Spemann Graduate School of Biology and Medicine, Freiburg, Germany (J. Ankerhold); University Medical Centre and Faculty of Medicine Freiburg, Freiburg (J. Ankerhold, S. Kessler, M. Schwemmle, K. Ciminski); Friedrich-Loeffler-Institut, Greifswald–Insel Riems, Germany (M. Beer)

**Keywords:** influenza, highly pathogenic avian influenza A(H5N1) viruses, H5N1 clade 2.3.4.4b, virus, MxA, zoonotic transmission, viruses, zoonoses, risk assessment

## Abstract

We show that human myxovirus resistance protein 1 (MxA) suppresses replication of highly pathogenic avian influenza A(H5N1) viruses isolated from mammals in vitro and in MxA-transgenic mice. However, H5N1 can evade MxA restriction through replacement of individual viral polymerase complex components from a human-adapted MxA-resistant strain in vitro.

Since 2022, clade 2.3.4.4b highly pathogenic avian influenza (HPAI) viruses of the H5N1 subtype have caused an increasing number of outbreaks in mammals worldwide (*1*). Since spring 2024, outbreaks of H5N1 clade 2.3.4.4b viruses have occurred in dairy cows in the United States, leading to the transmission of the virus to dairy farm workers, likely through close contact with infected cows or milk (*2*,*3*). Those events have raised concerns that H5N1 clade 2.3.4.4b viruses may further adapt to humans. Indeed, some current mammal H5N1 clade 2.3.4.4b isolates already carry adaptive mutations associated with enhanced binding to mammalian entry receptors, increased viral polymerase activity in mammalian cells, or escape from the recently identified BTN3A3 restriction factor (*1*,*2*,*4*). However, for sustained human-to-human transmission, HPAI H5N1 must overcome additional host barriers, including human myxovirus resistance protein 1 (MxA). 

MxA is an interferon-induced innate immune protein that suppresses replication of zoonotic influenza A viruses (IAVs) (*5*,*6*). Previous studies have demonstrated that human-adapted IAVs, such as the pandemic H1N1 virus A/Hamburg/4/2009 (pH1N1), evade MxA restriction through adaptive amino acids in the viral nucleoprotein (NP) (*7*). In contrast, MxA escape-mediating amino acids are absent in avian IAVs, such as the human HPAI H5N1 isolate A/Thailand/1(KAN-1)/2004 and the current mammal H5N1 clade 2.3.4.4b isolates (Appendix Figure). We used a risk assessment approach to investigate whether human MxA restricts zoonotic infections with mammalian H5N1 clade 2.3.4.4b isolates.

We determined the antiviral activity of MxA against HPAI H5N1 clade 2.3.4.4b A/blue fox/Finland/2023AI06876_071/2023 (blue fox H5N1) and A/white mink/Finland/2023AI08543_363/2023 (white mink H5N1) isolated from fur farms in Finland, A/cat/Poland/2023AI06401/2023 isolated from a fatally infected domestic cat in Poland (cat H5N1), and A/bovine/Texas/24–029328–01/2024 (bovine H5N1) isolated from a dairy cow in Texas, United States. Human pH1N1 and the prototypical H5N1 HPAI KAN-1 isolated from a human served as controls. 

Consistent with the lack of MxA resistance-mediating amino acids in NP (Appendix Figure), viral growth of KAN-1 and all H5N1 clade 2.3.4.4b isolates was suppressed in MDCK cells overexpressing antivirally active human MxA (MDCK-MxA), whereas the viruses replicated to peak titers of 5 × 10^6^ to 4 × 10^8^ PFU/mL in cells expressing the inactive MxA_T103A_ variant (MDCK-MxA_T103A_) (Figure 1). Replication of pH1N1 was slightly decreased in MDCK-MxA cells (Figure 1). 

**Figure 1:**
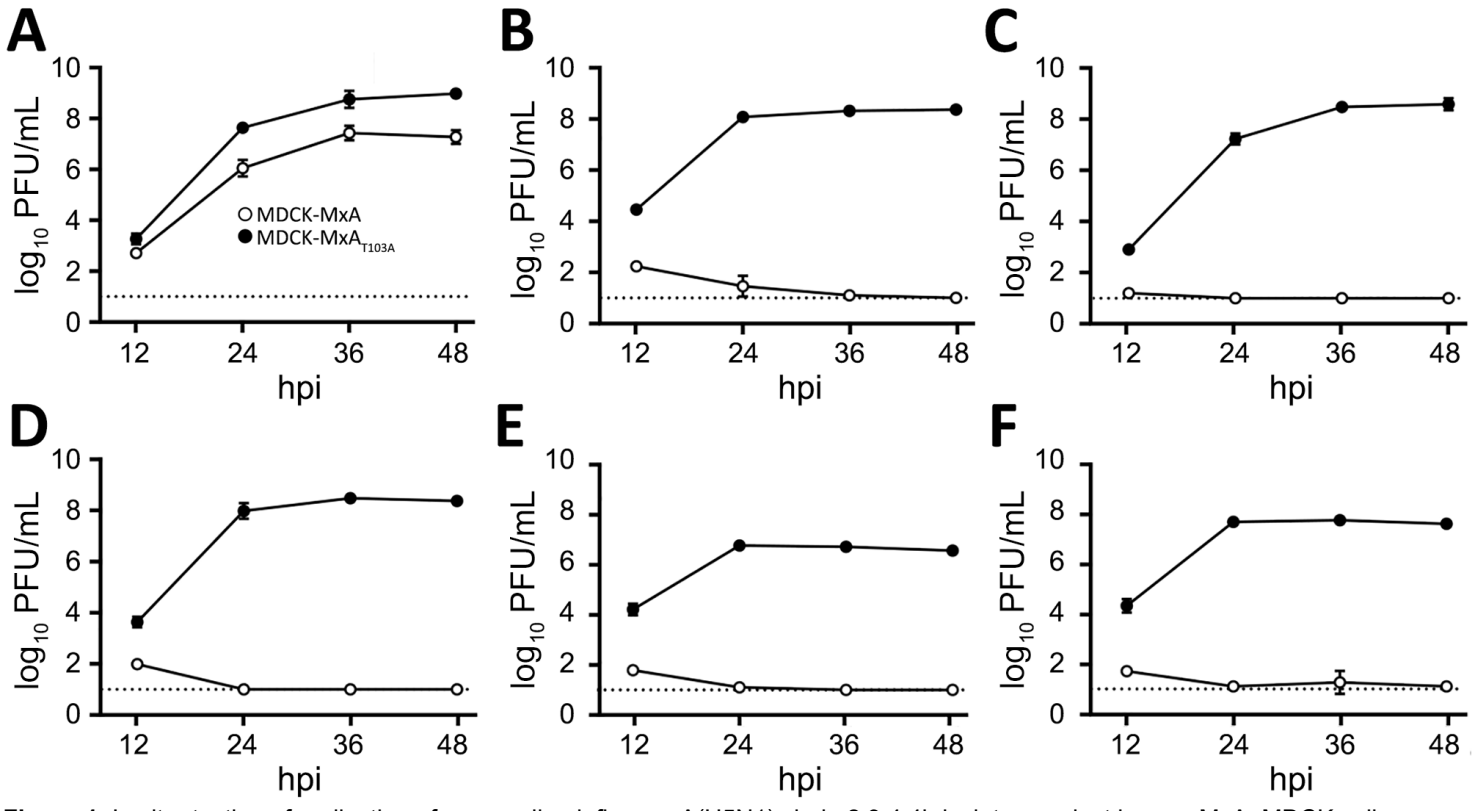
In vitro testing of replication of mammalian influenza A(H5N1) clade 2.3.4.4b isolates against human MxA. MDCK cells overexpressing MxA (MDCK-MxA) or antivirally inactive MxA_T103A_ (MDCK-MxA_T103A_) were infected with an influenza A isolate at a multiplicity of infection of 0.001; viral titers were determined at the indicated time points. A) Pandemic H1N1. B) KAN-1 H5N1. C) White mink H5N1. D) Blue fox H5N1. E) Cat H5N1. F) Bovine H5N1. Data are mean ± SD of n = 3 independent experiments. Dashed line indicates detection limit. hpi, hours postinfection; MxA, human myxovirus resistance protein 1.

To assess the importance of MxA in controlling zoonotic HPAI H5N1 infections in vivo, we intranasally inoculated C57BL/6 (B6) mice that lacked a functional Mx protein, as well as human MxA–transgenic mice (hMxA^tg/tg^), with pH1N1, KAN-1, or mammalian H5N1 clade 2.3.4.4b isolates (Appendix Figure). At 3 days postinfection, pH1N1 reached similar lung titers in B6 and hMxA^tg/tg^ mice, whereas viral replication of KAN-1 was >3,000-fold lower in the lungs of hMxA^tg/tg^ mice than in B6 mice. Of interest, viral replication of mammalian H5N1 clade 2.3.4.4b isolates was reduced 10-fold to 100-fold in hMxA^tg/tg^ mice, depending on the strain used (Appendix Figure). Despite those differences, infected hMxA^tg/tg^ mice appeared clinically healthy without any signs of disease, compared with infected B6 mice, which exhibited lethargy, ruffled fur, and a hunched posture.

In the past, some pandemic IAV strains overcame MxA restriction by reassortment of avian IAV surface proteins with mammal-adapted NP or polymerase components (*8*). We assessed whether replacement of NP or one of the polymerase components (polymerase basic [PB] 1 or 2 or polymerase acidic [PA]) would render the otherwise MxA-sensitive H5N1 HPAI polymerase complex MxA-resistant. After reconstituting bovine H5N1 polymerase complex with bovine H5N1 NP in the presence of MxA, we observed a strong suppression of the viral polymerase activity (Figure 2, panel A). However, we no longer detected that inhibitory effect of MxA when bovine H5N1 polymerase was reconstituted with pH1N1 NP. Conversely, the MxA-resistant pH1N1 polymerase complex was rendered MxA sensitive when reconstituted with the bovine H5N1 NP. Individual exchange of the remaining polymerase components showed that MxA restriction was partially overcome by a human-adapted PA but not by PB2 or PB1 (Figure 2, panel A). However, in contrast to NP (Appendix Figure), no MxA resistance amino acids are known in PA. Finally, because partial MxA resistance can be acquired in intermediate hosts expressing antivirally active Mx1 proteins (*7*,*9*), we determined the antiviral activity of cow, swine, and ferret Mx1 proteins against different viral polymerase complexes. Using HPAI H5N1 virus polymerase complexes, we observed a suppressive effect of bovine and, to some extent, swine Mx1 that was not observed for the MxA-resistant pH1N1 polymerase complex. Ferret Mx1 had no antiviral activity (Figure 2, panel B).

**Figure 2:**
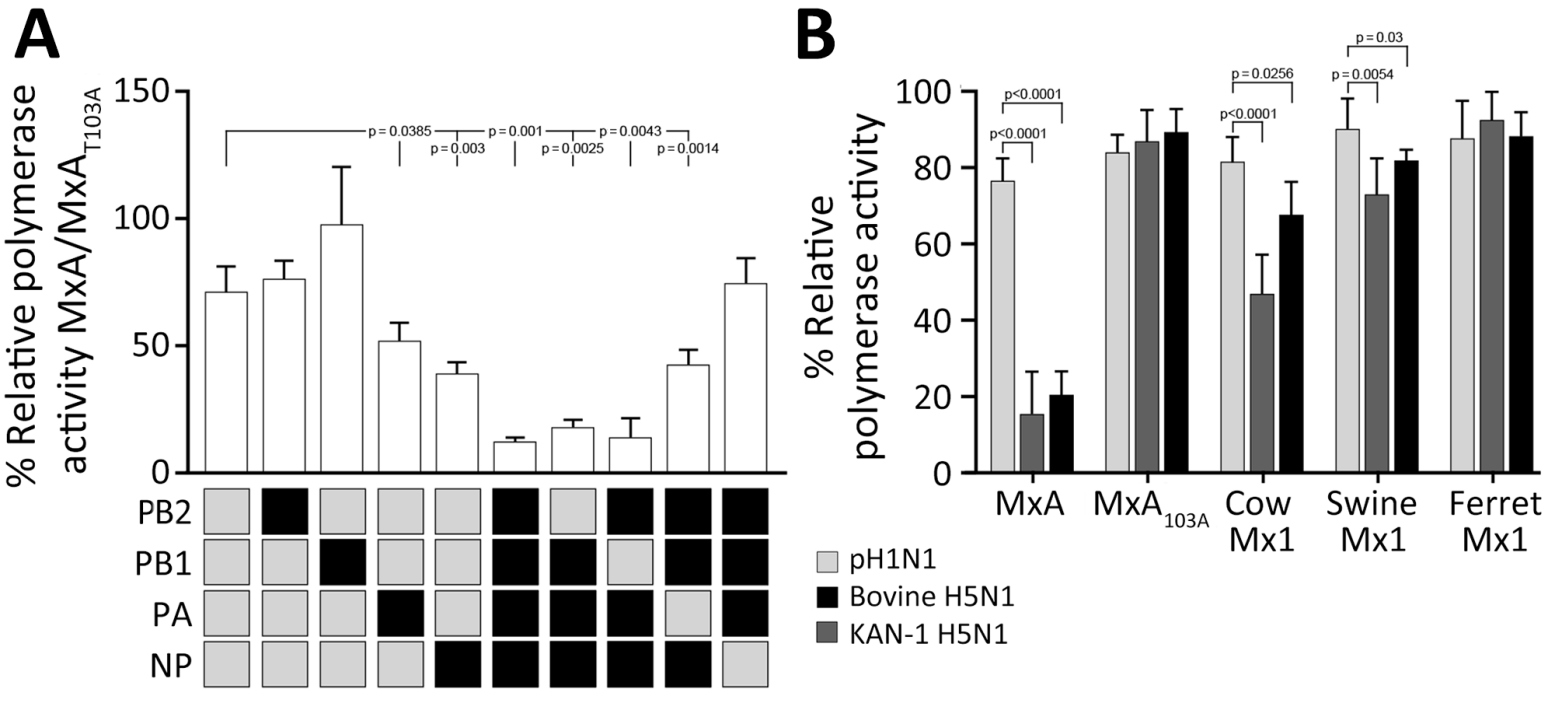
In vitro testing of bovine influenza A(H5N1) restriction through replacement of individual viral polymerase complex components from a human-adapted MxA-resistant strain. A) HEK293T cells were transfected with expression plasmids encoding the indicated pH1N1 or bovine H5N1 polymerase subunits PB2, PB1, PA, and NP together with expression plasmids encoding antivirally active MxA or the inactive MxA_T103A_ variant. After 24 hours, we determined the relative polymerase activity as the ratio of MxA to MxA_T103A_. Data are mean ± SD of n = 4 independent experiments. B) HEK293T cells were transfected with expression plasmids encoding the pH1N1, KAN-1 H5N1, or bovine H5N1 polymerase subunits PB2, PB1, and PA together with the respective NP. After 24 hours, we determined the polymerase activity in presence of the indicated MxA/Mx1 variant, normalized to a transfection control, and calculated relative to the empty vector control. Data are mean ± SD of n = 4 independent experiments. We used 2-tailed *t*-tests for statistical analysis. MxA, human myxovirus resistance protein 1; NP, nucleoprotein; PA, polymerase; PB, polymerase basic; pH1N1, pandemic H1N1.

Our data show that human MxA restricts current mammalian H5N1 clade 2.3.4.4b isolates. However, because this MxA-mediated restriction was less pronounced in hMxA^tg/tg^ mice, we speculate that adaptations in the viral polymerase, including PB2_E627K_ and PB2_M631L_ within the PB2 627 domain, have enabled the viruses to partially outpace MxA-mediated restriction (*10*). Given the ongoing circulation of bovine H5N1 in dairy cattle expressing antivirally active Mx1, increased surveillance could identify the potential emergence of MxA escape variants and provide early warning for possible future human-to-human transmission of these viruses.

Appendix Additional information about replication restriction of HPAI A(H5N1) clade 2.3.4.4b viruses by human immune factor, 2023–2024.
