# Supplementary material for: Replication Restriction of Influenza A(H5N1) Clade 2.3.4.4b Viruses by Human Immune Factor, 2023–2024
Source: Emerg Infect Dis. 2025 Jan;31(1):199–202. doi: 10.3201/eid3101.241236 (PMC11682815; doi:10.3201/eid3101.241236)
Supplement: Appendix — Additional information about replication restriction of HPAI A(H5N1) clade 2.3.4.4b viruses by human immune factor, 2023–2024. [file 24-1236-Techapp-s1.pdf]

Article DOI: https://doi.org/10.3201/eid3101.241236

EID cannot ensure accessibility for supplementary materials supplied by authors. Readers who have difficulty accessing supplementary content should contact the authors for assistance.

Replication Restriction of HPAI A(H5N1) Clade 2.3.4.4b Viruses by Human Immune Factor, 2023–2024

Appendix

Virus

The human-adapted A/Hamburg/4/2009 (pdmH1N1) (GenBank accession nos. GQ166207, GQ166209, GQ166211, GQ166213, GQ166215, GQ166217, GQ166219, and GQ166221) and the zoonotic A/Thailand/1(KAN-1)/2004 (H5N1) viruses (GenBank accession nos. CY111595 to CY111602) were available in our virus collection. The clade 2.3.4.4b H5N1 isolates A/blue fox/Finland/2023AI06876_071/2023 (GISAID isolate ID: EPI2701776), A/white mink/Finland/2023AI08543_363/2023 (GISAID isolate ID: EPI2791006), A/cat/Poland/2023AI06401/2023 (GISAID isolate ID: EPI2610691), and A/bovine/Texas/24–029328–01/2024 (GenBank accession nos. PQ106994 to PQ107001) were propagated in embryonated SPF-chicken eggs at 37°C. At 5 days post infection, the allantoic fluid was harvested and used as virus stock. Virus stocks were sequenced and stock titers were determined by a plaque assay on MDCKII cells.

Cell Lines

HEK293T cells were obtained from the American Type Culture Collection (ATCC; CRL-3216). Madin-Darby Canine Kidney (MDCK) type II cells were purchase from Merck (catalog number 00062107). MDCK-MxA and MDCK-MxA_{T103A} were obtained from Jesse D. Bloom (Fred Hutchinson Cancer Research Center, United States) (*1*). All cell lines were cultured in Dulbecco’s modified Eagle’s medium (DMEM, GIBCO, Thermo Fisher Scientific) containing

10% fetal calf serum (FCS), 100 U penicillin and 100 µg streptomycin mL^{-1} at 37 °C and 5% CO_2.

Virus Infections

MDCK-MxA and MDCK-MxA_{T103A} cells were seeded and grown in 6-well plates. Prior to infection cells were washed with phosphate buffered saline (PBS) containing 0.2% bovine serum albumin (BSA) and then infected with the indicated virus at an MOI of 0.001 in infection medium (DMEM, containing 0.2% BSA and 100 U penicillin and 100 µg streptomycin μL^{-1}). For pdmH1N1 1 µg mL^{-1} TPCK-treated trypsin was added into the infection medium. Viral titers were determined by plaque assay on MDCK II cells.

Polymerase Reconstitution Assay

HEK293T cells were seeded and grown in 12-well plates and transfected with pCAGGs expression plasmids encoding the polymerase subunits PB2, PB1 and PA (each 10 ng) together with 100 ng of pCAGGs plasmids coding for NP using Lipofectamine 2000 (Thermo Fisher Scientific, 11668019) according to the manufacturer's protocol. The firefly luciferase-encoding construct pPolI-FFLuc-RT (100 ng) served as a viral minigenome. Transfection efficiency was determined by co-transfecting 30 ng of the pRL-SV40 plasmid coding for the *Renilla* luciferase. Additionally, 200 ng of pCAGGs plasmids expressing human MxA, the inactive MxA_{T103A} construct, or one of the Mx1 homologs from *Bos taurus* (cow Mx1), *Sus scrofa domesticus* (swine Mx1), or *Mustela putorius furo* (ferret Mx1) that were obtained from Georg Kochs (University Medical Centre and Faculty of Medicine Freiburg, Freiburg, Germany) (*2*), or an empty vector. At 24 hours post transfection cells were lysed and the firefly and *Renilla* luciferase activities were measured by using the Dual-Luciferase® reporter assay system (Promega). Firefly luciferase activity was normalized to the Renilla luciferase activity. The empty vector control was used for calculation of the relative polymerase activity.

Western Blot

To determine protein levels, samples were incubated at 95°C in Laemmli buffer and subsequently separated by sodium dodecyl sulfate polyacrylamide gel electrophoresis (SDS–PAGE). Separated protein samples were blotted onto a nitrocellulose membrane. Proteins were

detected using specific antibodies against the highly conserved G domain in MxA (M143)23, NP (produced in-house; 1:1,000), or actin (Sigma-Aldrich, A2228; 1:1,000), respectively. Primary antibodies were detected using IRDye® secondary antibodies (LICORbio, 926–32210 and 926–68021, 1:10,000).

Mouse Infections

All mouse experiments were performed in accordance with the guidelines of the German animal protection law and were approved by the state of Baden-Württemberg (Regierungspräsidium Freiburg; reference number: 35–9185.81/G-24/034). C57BL/6 (B6) mice were obtained from Janvier and hMxA$^{tg/tg}$ mice were bred in-house at the Institute of Virology, Freiburg. All mice were housed in individually ventilated cages (Green Line, Tecniplast) with stable bedding (2HK: TAPVEI® BEDDING), at a temperature of 20.2 ± 1.1°C, a humidity of 55%±7.1% and a 12 h:12 h light:dark cycle. The mice were fed ad libitum with food made from natural ingredients (Germany, altromin 1314). All animal work was performed in BSL3 containment facilities. For infection experiments, sex-and age-matched mice were anaesthetized by isoflurane inhalation and were subsequently inoculated intranasally with 40 µL of the indicated virus dose diluted in Opti-MEM containing 0.3% BSA. Throughout the experiment, mice were monitored daily for changes in bodyweight and other signs of disease. At 3 dpi mice were sacrificed and the lung was dissected. Organs were homogenized in 800 µL PBS by three subsequent rounds of mechanical treatment for 25 s each at 6.5 ms^{-1}. Tissue debris was removed by centrifuging homogenates for 5 min at 5,000 rpm at 4°C and samples were stored at −80°C until further processing. Viral organ titers were determined by plaque assay.

References

1. Ashenberg O, Padmakumar J, Doud MB, Bloom JD. Deep mutational scanning identifies sites in influenza nucleoprotein that affect viral inhibition by MxA. PLoS Pathog. 2017;13:e1006288. PubMed https://doi.org/10.1371/journal.ppat.1006288

2. Fuchs J, Oschwald A, Graf L, Kochs G. Tick-transmitted thogotovirus gains high virulence by a single MxA escape mutation in the viral nucleoprotein. PLoS Pathog. 2020;16:e1009038. PubMed https://doi.org/10.1371/journal.ppat.1009038

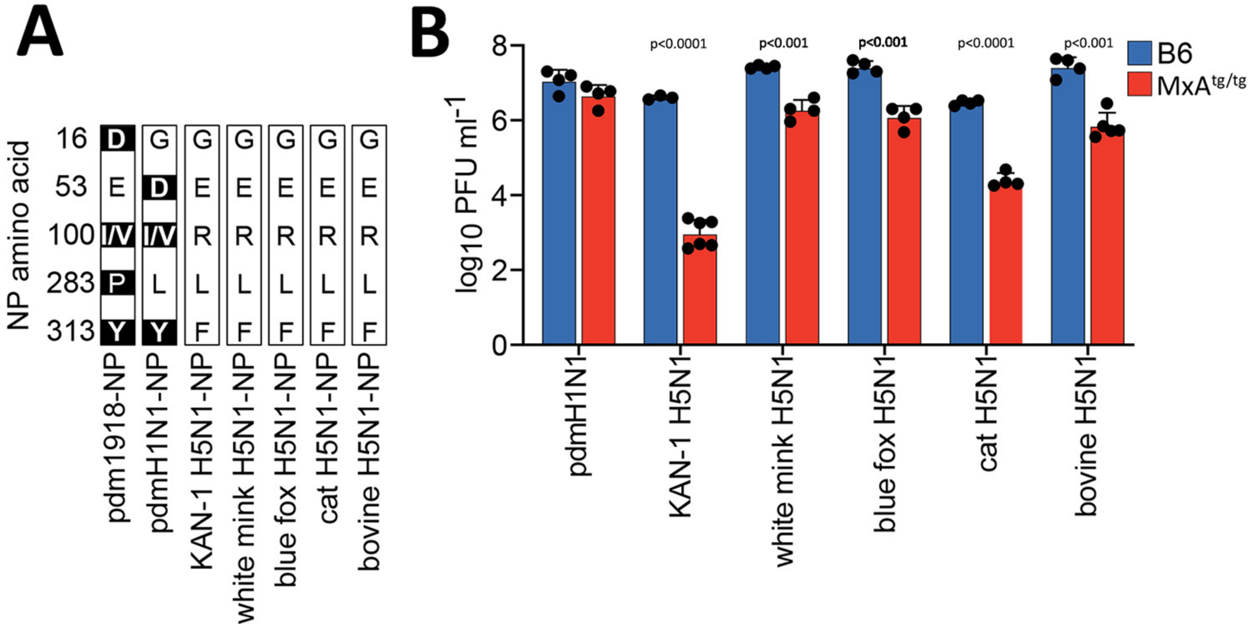

Appendix Figure. A) MxA resistance amino acids in NP from the human 1918 pandemic strain (pdm1918) and pdmH1N1 strain are highlighted. The prototypical avian H5N1 virus KAN-1 and the mammalian H5N1 clade 2.3.4.4b isolates white mink H5N1, blue fox H5N1, cat H5N1, or bovine H5N1 lack any of the known MxA-resistance amino acid residues. B) hMxA$^{tg/tg}$ (n = 3 or 4) or wild-type B6 mice (n = 4 or 6) were inoculated with 1×10^{4} PFU of the indicated virus. Lung viral titers were determined 3 dpi. Data are mean ± SD. Statistical analysis was performed using 2-tailed t-tests; ***p = 0.001; ****p = 0.0001.
